# CVD Deposited Epoxy Copolymers as Protective Coatings for Optical Surfaces

**DOI:** 10.3390/polym15030652

**Published:** 2023-01-27

**Authors:** Merve Karabıyık, Gizem Cihanoğlu, Özgenç Ebil

**Affiliations:** Department of Chemical Engineering, İzmir Institute of Technology, 35430 Urla, Turkey

**Keywords:** copolymer thin film, protective coatings, poly(GMA), poly(V4D4), optical surfaces, iCVD

## Abstract

Copolymer thin films of glycidyl methacrylate (GMA), ethylene glycol dimethacrylate (EGDMA) and 2,4,6,8-tetramethyl-2,4,6,8-tetravinylcyclotetrasiloxane (V4D4) were synthesized via initiated chemical vapor deposition (iCVD) as protective coatings for optical surfaces. Chemical durability in various solvents, corrosion resistance, adhesion to substrate, thermal resistance and optical transmittance of the films were evaluated. Crosslinked thin films exhibited high chemical resistance to strong organic solvents and excellent adhesion to substrates. Poly(GMA-co-EGDMA) and poly(GMA-co-V4D4) copolymers demonstrated protection against water (<1% thickness loss), high salt resistance (<1.5% thickness loss), and high optical transparency (~90% in visible spectrum) making them ideal coating materials for optical surfaces. Combining increased mechanical properties of GMA and chemical durability V4D4, the iCVD process provides a fast and low-cost alternative for the fabrication of protective coatings.

## 1. Introduction

Optical materials (glasses, mirrors, lenses, prisms, filters, etc.) are widely used in a variety of applications such as electronic and medical equipment, automotive and construction sector, aerospace industries, and various military and civilian electro-optic devices. Due to their widespread use, these materials may work in very harsh, unstable and corrosive conditions, and can be exposed to various solvents, dust and humidity, vibration, radiation, rapid temperature changes and physical abuse. Such conditions can reduce the performance and useful lifespan of these materials [1,2,3,4]. Repairing damaged optical surfaces is usually expensive if not impossible, and protection of the materials is thus essential. 

There are two basic requirements for coatings on optical surfaces. The coating material(s) should not interfere with the optical performance of the system, i.e., the coatings should be transparent in the respective wavelength ranges, and the coating should provide acceptable chemical and/or physical protection. In this regard, polymer films have attracted a lot of interest since they can provide physical and chemical protection to optical surfaces [2]. In the literature, a variety of polymeric materials such as poly(methyl methacrylate) [5], poly(carbonate) [6,7], poly(styrene) [8], poly(urethane) [9], poly(ethylene terephthalate), poly(ethylene naphthalate) [10], benzocyclobutene [11], perfluorovinyl ether cyclopolymer (CY-TOP) [12], tetrafluoroethylene and perfluorovinyl ether copolymer (Teflon AF) [13], fluorinated poly(arylene ether sulfide) [14], and fluorinated hyperbranched polymers [15] were investigated as coatings on optical surfaces. Most of these studies suggest that homopolymer films do not exhibit thermal and mechanical strength required for the protection of optical surfaces on their own [16,17,18]. To overcome this issue, crosslinking and combining polymeric materials [18,19,20,21] or modifying the polymer surface with inorganic coatings to improve thermal, mechanical and optical properties and adhesion between the coating and the substrate [16,17,22] have been evaluated. Zhao et al. fabricated liquid-release polymeric gel films with a novel bilayer structure which consist of a slippery liquid-locked rough top layer and liquid-supplied bottom layer by one-pot casting [19]. Bhattacharjee et al. fabricated polymeric sheets by combining cationic amphiphilic water-soluble polyethylenimine derivative (QPEINH-C6) and poly(vinyl alcohol) (PVA). Polymeric sheets showed antimicrobial properties and high mechanical durability and were highly transparent (86−90% transmittance in visible spectrum) [20]. Sulfoxide biphenyl polyimide PI (TFSODA/BPDA) optical coatings prepared via a low-temperature process demonstrated high optical performance (88.5% between 380 and 780 nm) and good thermal properties [21]. A polyimide composite membrane with SiO_2_ antireflective layer prepared by a sol-gel method exhibited good thermal stability and up to 93% optical transmittance between 500 and 800 nm [17]. Sun et al. combined oxygen plasma and pulse laser deposition to fabricate polycarbonate with nanoporous silica film. Silica coated polymer film exhibited high transparency (89.9% within 420–700 nm), excellent mechanical robustness, and excellent antifogging performance [16]. Althues et al. fabricated transparent nanocomposites consisting of europium doped yttrium vanadate (YVO4: Eu) nanoparticles in methyl methacrylate (MMA) and lauryl acrylate (LA) matrices with excellent optical properties (transmission >90% in visible spectrum) [22].

The commonly used methods for fabrication of polymeric films such as dip coating and spin coating are usually applied to flat surfaces, but issues related to solvent use (wetting of surface, solvent evaporation, etc.) and difficulty in accurate control of film thickness still exist. In addition, optical materials to be coated may be damaged due to substrate-solvent interactions. Since optical surfaces also vary greatly in shape and size, and may have micro-and nano-structured features, solventless processes such as physical vapor deposition (PVD) and chemical vapor deposition (CVD) should be considered despite a higher cost of fabrication due to vacuum requirements [2,23]. A CVD process is similar to a PVD but at least one chemical reaction takes place for the deposition of the thin film [24]. Initiated chemical vapor deposition (iCVD) is a well-established process for synthesis of thin polymeric films. Compared to other CVD methods, iCVD offers a lower thermal budget (low filament temperatures between 200–400 °C). The low deposition temperature enables use of a variety of substrates including plastics, inorganic materials, textiles, glass, membranes, carbon nanotubes, etc. The iCVD process can achieve high deposition rates with precise thickness and morphology control and without damaging functional groups of monomers [25,26,27,28,29,30,31,32,33,34,35,36,37,38]. During iCVD polymerization, monomer units are sent to the reactor in the vapor phase where they are adsorbed on the cooled substrate surface (0–40 °C). The unsaturated bonds of the adsorbed monomers are activated by free radicals produced via thermal decomposition of an initiator molecule (typically 200–400 °C). Free radical polymerization takes place (including initiation, propagation and termination steps) on the substrate surface [25,26,27,29,33,37,39,40,41,42,43,44,45]. In recent years, iCVD has emerged as an attractive alternative for fabrication of polymer thin films to protect surfaces against aggressive media. Employing a transparent and very adhesive polymer thin film such as poly(glycidyl methacrylate) (poly(GMA)) can lead to enhanced chemical and mechanical properties due to the epoxy ring (-C_2_H_3_O) which provides a good binding site [29,46,47]. In addition, poly(GMA) is a promising candidate for coatings on optical surfaces requiring transparent and durable protective layers [48,49]. 

Here, we report the fabrication of robust cross-linked copolymer thin films of GMA with ethylene glycol dimethacrylate (EGDMA) and 2,4,6,8-tetramethyl-2,4,6,8-tetravinylcyclotetrasiloxane (V4D4) via iCVD as protective coatings for optical surfaces. Cross-linked copolymers can improve chemical and mechanical durability while maintaining high optical transmittance in the visible spectrum. To the best of our knowledge, this is the first study in the literature where the effect of cross-linkers on poly(GMA) based polymer thin films are investigated as protective coatings. This work also demonstrates the feasibility of non-fluorinated polymers to protect optical surfaces. Another goal of this contribution is to demonstrate the effectiveness of the iCVD process to tailor properties of polymer films by crosslinking during polymerization without losing functional groups. 

## 2. Materials and Methods

### 2.1. Materials

Homopolymers of glycidyl methacrylate (poly(GMA)), ethylene glycol dimethacrylate (poly(EGDMA)) and 2,4,6,8-tetramethyl-2,4,6,8-tetravinylcyclotetrasiloxane (poly(V4D4)), and copolymers poly(GMA-co-EDMA) and poly(GMA-co-V4D4) were fabricated as thin films on crystalline silicon (c-Si) and glass substrates. Analytical grade chemicals, GMA (Sigma Aldrich, USA, 97%) as monomer, EGDMA (Sigma Aldrich, Burlington, MA, USA, 98%) and V4D4 (SigmaAldrich, USA, 97%) as crosslinkers and tert-butyl peroxide (TBPO, Sigma Aldrich, USA, 98%) as the initiator were used for the synthesis of thin film coatings. Various organic solvents such as toluene (Sigma-Aldrich, ≥99.5%), dichloromethane (DCM, Sigma Aldrich, USA), ethanol (Sigma Aldrich, USA, ≥99.8%), 1-propanol (Sigma-Aldrich, USA, ≥99.8%), acetone (Sigma Aldrich, USA, ≥99.5%), tetrahydrofuran (THF, Sigma Aldrich, USA, 99.9%), and N,N-dimethylformamide (DMF, Sigma Aldrich, USA) were used for the chemical durability tests.

### 2.2. Fabrication of Polymer Coatings

Polymer coatings were fabricated using a custom-built iCVD system. The square reactor chamber was 32 cm by 32 cm in size and had a height of 4 cm. A nichrome filament array suspended 2.5 cm above the substrate was used to provide thermal energy for the decomposition of the initiator. The filament temperature was measured with a type-K (Omega Engineering) thermocouple attached to the filaments. The temperature of the substrate was controlled by an external circulator (WiseCircu WCR-P8) connected to the bottom of the reactor. The vacuum was provided via a rotary vane pump (BSV10, Baosi). The reactor pressure was controlled with a throttling butterfly valve (MKS Model 253) connected to a pressure controller (MKS Type 651C). Poly(GMA), poly(EGDMA), poly(V4D4) homopolymers and poly(GMA-co-EGDMA) and poly(GMA-co-V4D4) copolymers were deposited on crystalline silicon (c-Si) wafers and BK7 optical glass substrates. In order to obtain sufficient vapor pressure, GMA, EGDMA and V4D4 monomers were heated to 65, 85 and 90 °C, respectively. For homopolymer coatings, monomers were fed into the chamber through a special mass-flow controller (MKS1150C). TBPO was metered into the reactor at room temperature through a mass-flow controller (MKS 1479A). Reactor pressure was maintained at 250 or 515 mTorr during this study. The average thickness of polymer coatings was 350 nm ± 50 nm. The deposition conditions for homopolymers and copolymers are summarized in Table 1.

### 2.3. Film Characterization

Mprobe-Vis20 reflectometer system with a spectral range of 400–1100 nm and 2 nm measurement accuracy was used to measure the thicknesses of fabricated films. For the evaluation of the quality and chemical composition of fabricated polymer films a Perkin Elmer Inc. (Shelton, CT) BX FTIR (Fourier Transform Infrared Spectroscopy) Spectrometer was used. The spectra of the polymer films were measured from 4000 to 650 cm^−1^. All spectra were baseline corrected and thickness normalized. Surface morphologies of fabricated iCVD polymer coatings were investigated using a Scanning Electron Microscope (SEM) (FEI Quanta250, Hillsboro, OR). Surface roughness was measured by Atomic-force Microscopy (AFM) using a Nanosurf-Flex Axiom system. A BEL MPL-2 polarization microscope was employed to evaluate the surface of the films before and after the adhesion tests. Thermogravimetric Analysis (TGA) was performed using a Shimadzu TGA-51 system. 

### 2.4. Chemical Stability and Durability Tests

The durability of fabricated homopolymer and copolymer coatings was investigated by solubility, adhesions, and thermal tests. Solubility tests were performed by immersing coatings into various solvents for 30 min. The immersed samples were then dried at 70 °C for 1 h to remove excess solvent from the surface. Salt resistance tests were performed in 5 wt. % NaCl solutions at room temperature for 1 day. Film thicknesses were measured before and after the immersion. The experiments were carried out at least three times, and the standard deviation was ± 5%. The adhesion tests of polymer coatings to silicon substrates were performed by following the procedure described previously in the literature [50,51]. A cellophane adhesive tape was placed on the film surface and then rapidly removed from the surface at the angle that was normal to the surface. The percentage of delamination was calculated by dividing the test surface into equally spaced grids. The coating adhesion was evaluated by optical microscope analysis. The thermal durability was evaluated by annealing the coatings at temperatures up to 250 °C for 4 h (10 °C min^−1^ heating rate) in a furnace. Thermogravimetric analysis of samples (ca. 10 mg) was performed at a constant heating rate of 10 °C min^−1^, from room temperature to 1000 °C, under a nitrogen flow.

## 3. Results and Discussion

### 3.1. Deposition Rate

**Figure 1:**
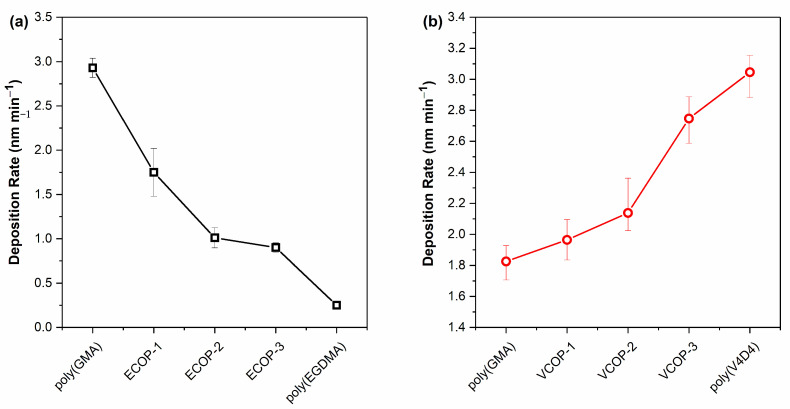
Deposition rates of (**a**) poly(GMA), poly(EGDMA) and ECOP (black squares), and (**b**) poly(GMA), poly(V4D4) and VCOP (red circles).

For copolymer depositions, the flow rates of monomers were varied to obtain copolymers with different compositions, as shown in Table 1. Although the iCVD process can achieve extremely high deposition rates (of the order of nm min^−1^), the process parameters were carefully selected to yield much lower deposition rates (<10 nm min^−1^) to accurately control film thickness and uniformity. In the iCVD process, the deposition rate is inversely proportional to the substrate temperature. Lower substrate temperature leads to increased monomer adsorption on the surface resulting in a higher deposition rate [30,31]. However, a low substrate temperature might also lead to condensation due to excessive monomer concentration on the substrate surface [25]. For the deposition of poly(GMA-co-V4D4) (VCOP) copolymer, the substrate temperature was increased to 35 °C from 25 °C to avoid condensation. Poly(GMA-co-EGDMA) (ECOP) copolymer depositions were performed at 25 °C. Copolymer deposition rates for ECOP and VCOP copolymers are shown in Figure 1. The deposition rate of poly(GMA-co-EGDMA) copolymers decreased as the flow rate ratio of EGDMA/GMA increased from 0.25 to 1. For the poly(GMA-co-V4D4) copolymers, higher deposition rates were observed as the flow rate ratio of V4D4/GMA increased. 

### 3.2. Chemical Composition

**Figure 2:**
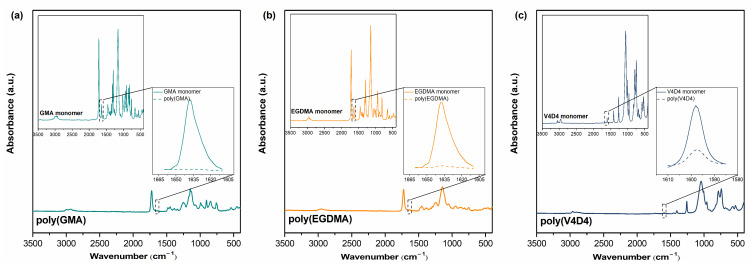
FTIR spectra of (**a**) GMA (green), (**b**) EGDMA (orange) and (**c**) V4D4 (blue) monomers and their polymers.

Chemical compositions of monomers and homopolymers were investigated via FTIR analysis as shown in Figure 2. The characteristic peaks of C-O-C epoxy ring vibrations at 758, 845 and 906 cm^−1^ are clearly shown in poly(GMA) [52]. The symmetric epoxy ring deformation, asymmetric epoxy ring deformation, and the epoxy ring breathing vibration for poly(GMA) are seen in the region of 840–853 cm^−1^, 912–920 cm^−1^, and near 1241–1251 cm^−1^, respectively. The strong absorption from C-O-C asymmetric and symmetric stretching vibrations in the region of 883–912 cm^−1^ and 1246–1322 cm^−1^ also masks the epoxy ring deformation and breathing vibrations, respectively [53]. The region of the infrared spectrum from 1200 to 700 cm^−1^ belongs to the fingerprint region. Many different vibrations, including C-O, C-C and C-N single bond stretches and C-H bending vibrations are found in this region, therefore stretching and bending vibrations of various groups overlap with that of the epoxy group [52,53]. For poly(EGDMA) homopolymer, the peak formed at 1716 cm^−1^ belongs to the valence vibration of the ester group (C=O) [54]. For poly(GMA) and poly(EGDMA) homopolymers, the peaks at 1161, 1252 and 1727 cm^−1^ show C-O, C-C and C=O stretching vibrations, respectively [55,56]. The band around 1630–1640 cm^−1^ in both monomer GMA and EGDMA represents the vinyl group and the absence of this peak in both homopolymer spectra indicates complete polymerization of all monomers on the substrate surface [57]. Figure 2c shows that the vinyl (CH_2_=CH–) group peak intensity of V4D4 monomer at 1598 cm^−1^ decreased to a certain extent in poly(V4D4) spectrum, indicating that polymerization of V4D4 monomer was successfully achieved by consuming vinyl functionality; however, a significant amount of vinyl group still remained in the final polymer. A V4D4 monomer molecule contains 4 vinyl groups and the complete consumption of the vinyl group by free radical polymerization was shown to be impossible due to the steric hindrance [58,59,60]. The existence of monomer-specific wagging of Si-(CH_2_)_x_-Si (963 cm^−1^), asymmetric Si-O-Si stretching (1075 cm^−1^), Si-CH_3_ symmetric bending (1260 cm^−1^), and the bending in Si-CH_2_ (1410 cm^−1^) in poly(V4D4) spectrum also indicate the preservation of functional groups of the monomer [58,59,60,61].

**Figure 3:**
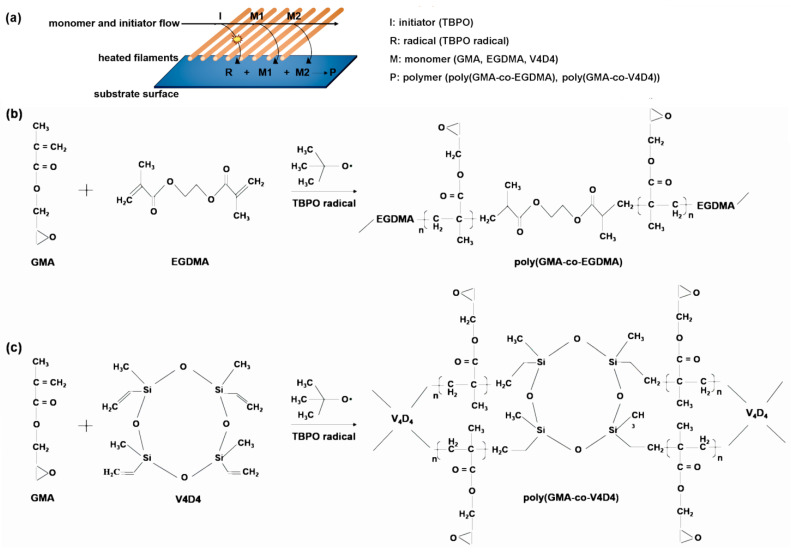
Schematics of (**a**) iCVD process, (**b**) poly(GMA-co-EGDMA) and (**c**) poly(GMA-co-V4D4) copolymer film synthesis.

**Figure 4:**
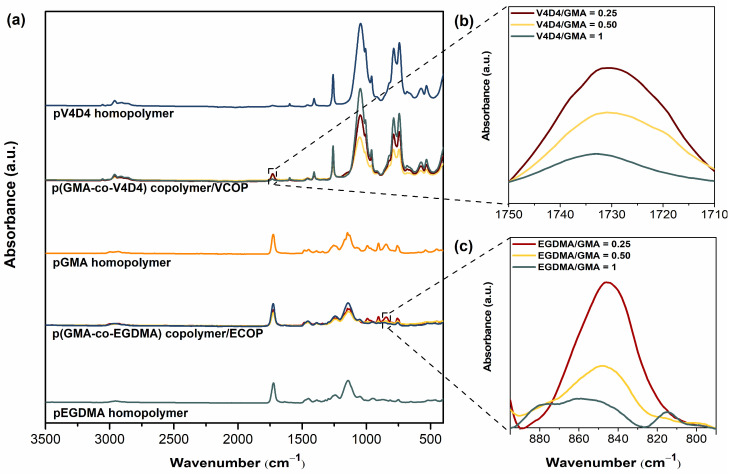
FTIR spectra of (**a**) GMA, EGDMA and V4D4 homopolymers and their copolymers, (**b**) poly(GMA-co-V4D4) copolymers, and (**c**) poly(GMA-co-EGDMA) copolymers.

Poly(GMA-co-EGDMA) and poly(GMA-co-V4D4) polymerizations in iCVD are shown in Figure 3. It should be noted that in Figure 3c, poly(GMA-co-V4D4) copolymer structure does not represent the real structure since not all vinyl bonds in V4D4 monomer participate in cross-linking, as discussed above. Figure 4 shows FTIR spectra of homopolymers and copolymers deposited via iCVD. Figure 4b shows the enlargement of a portion of Figure 4a emphasizing the absorption peak from 1710 and 1750 cm^−1^ where the specific peaks of GMA are located. Peak intensities related to C=O stretching decreased as the V4D4/GMA flow rate ratio was increased gradually from 0.25 to 1. Figure 4c shows the enlargements of a portion of Figure 4a, indicating the epoxy peak (the C-O-C vibration) of GMA at 846 cm^−1^. The composition of copolymer coatings can be controlled by adjusting the monomer flow rates. The highest peak intensity was observed when the EGDMA/GMA flow rate ratio was 0.25 and declined with decreasing GMA in copolymer composition. However, the decrease in the peak intensity (peak area) does not necessarily correspond to feed composition due to difference in monomer adsorption rates on the surface [25].

### 3.3. Chemical Stability

**Figure 5:**
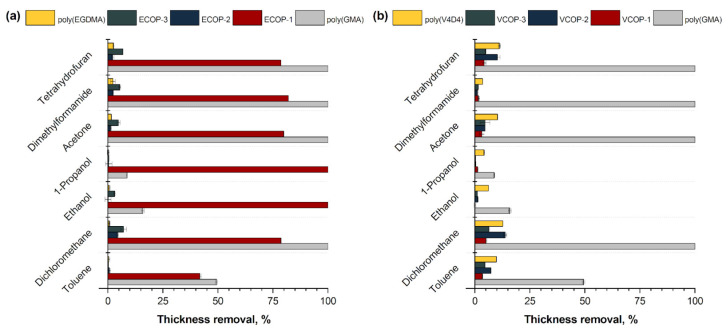
Relative change in film thickness of (**a**) poly(GMA), poly(EGDMA) homopolymers and their copolymers, and (**b**) poly(GMA), poly(V4D4) homopolymers and their copolymers in various solvents.

Chemical stability of homopolymers and copolymers was evaluated by immersion of samples in various organic solvents. The relative changes in thicknesses of homo and copolymer coatings before and after immersion in organic solvents (DCM, acetone, THF, DMF, IPA and ethanol) are shown in Figure 5. The Hildebrand solubility parameter (δ) is a good way to determine whether a substance is a good solvent or nonsolvent for a polymer [62]. When the difference between the Hildebrand solubility parameters of the polymer (δp) and solvent (δs) is low (|δp−δs| ≤ 2), the solvent may be considered a good solvent (or solvating solvent) of that polymer. On the other hand, when this difference is high (|δp−δs| ≥ 2), this solvent is considered a thermodynamically poor solvent of the polymer. According to the literature data, DCM can be considered as good (δs = 19.8) [63], and ethanol is a poor solvent (δs = 26.6) [63] of the epoxy polymer (δp = 17.8) [64]. Unlike poly(EGDMA) homopolymer, poly(GMA) homopolymer is not usually resistant to organic solvents. As expected, poly(GMA-co-EGDMA) copolymers with higher EGDMA content (ECOP-2 and ECOP-3) exhibited better resistance than poly(GMA) with less than 10% thickness loss. Poly(V4D4) homopolymer showed better resistance to organic solvents compared to poly(GMA) with a maximum 15% thickness loss. Among poly(GMA-co-V4D4) copolymer coatings, VCOP-1 demonstrated the best resistance against organic solvents with a maximum 5% thickness loss. The difference in resistivity to organic solvents between poly(GMA-co-V4D4) and poly(GMA-co-EGDMA) coatings is related to the difference in the number of reactive sites per monomer. V4D4 monomer provides two more reactive groups for cross-linking compared to EGDMA monomer. Therefore, it is expected that poly(GMA-co-V4D4) copolymers should be more resistant to organic solvents [61]. However, we did not observe substantial difference in performances of poly(GMA-co-V4D4) and poly(GMA-co-EGDMA) copolymers (except ECOP-1) which might be related to the partial polymerization of vinyl groups in V4D4 due to steric hindrance.

**Figure 6:**
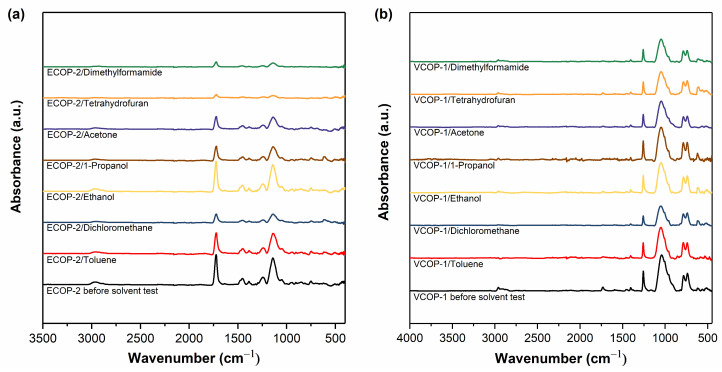
FTIR spectra of (**a**) ECOP-2 and (**b**) VCOP-1 copolymers before and after immersion in organic solvents.

FTIR analysis was repeated after immersion in solvents. Figure 6 shows FTIR spectra of ECOP-2 and VCOP-1 copolymer coatings which were found to be the most durable copolymer films in organic solvents. All characteristic peaks of poly(GMA-EGDMA) and poly(GMA-co-V4D4) copolymers remained visible after immersion in THF, DCM, DMF, acetone, ethanol, toluene, and 1-propanol for 30 min. However, the reduction in peak intensities was much less for ECOP-2 and VCOP-1 compared to other copolymers. VCOP-1 exhibited very slight peak intensity reduction in only DCM which was one of the strongest solvents used in this study.

**Figure 7:**
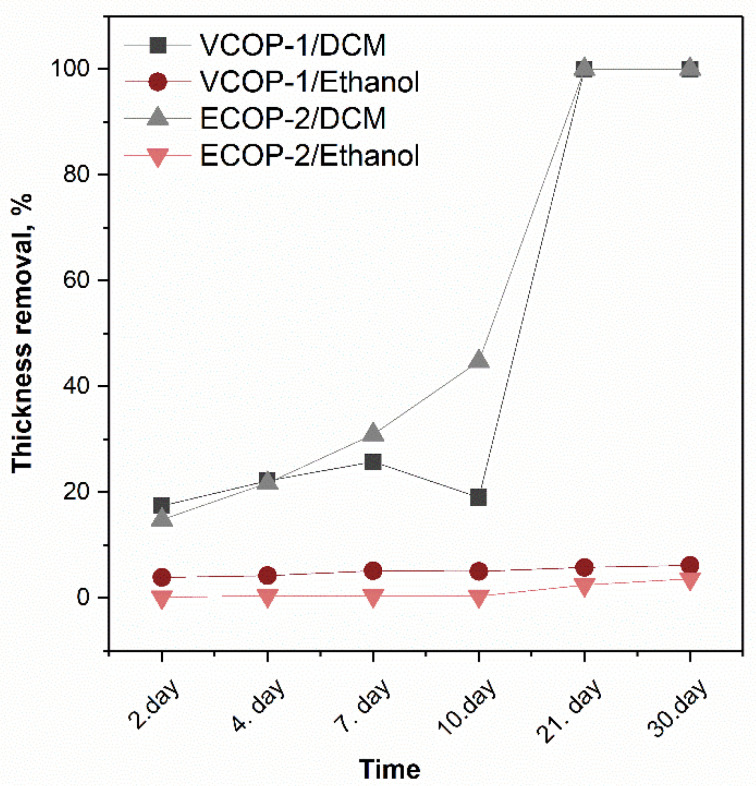
Relative change in film thickness for copolymers after immersion in DCM and ethanol.

To further evaluate the chemical durability of ECOP-2 and VCOP-1 copolymer coatings, samples were immersed in DCM and ethanol (a weaker solvent) for 30 days. The relative changes in film thicknesses after 30 days are shown in Figure 7. Immersion in ethanol did not lead to a significant thickness loss for the copolymers. However, ECOP-2 and VCOP-1 coatings were dissolved in DCM completely after 20 days. Still, the results indicate that these copolymers can act as effective protective coatings against accidental exposure to very strong solvents and can serve as protective barriers very long times in weak solvents.

**Figure 8:**
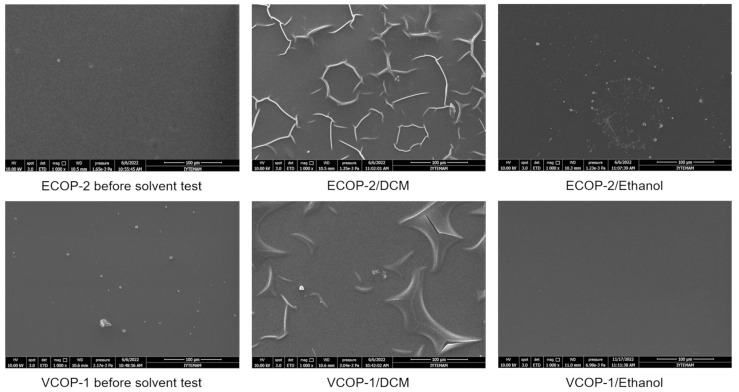
SEM images of ECOP-2 and VCOP-1 copolymers before and after immersion in DCM and ethanol for 30 min.

SEM images of ECOP-2 and VCOP-1 copolymer coatings before and after immersion in DCM and ethanol are shown in Figure 8. ECOP-2 copolymer showed a slight increase in surface roughness after immersion in ethanol while VCOP-1 copolymer showed no change in surface morphology. Both copolymers showed wrinkling leading to crack formation, and eventually complete delamination from the surface after immersion in DCM. It is suspected that immersion in DCM solvent weakens the adhesion of coatings to c-Si substrate and coatings are released from the substrates in pieces. The difference in the amount of delamination for ECOP-2 and VCOP-1 coatings can be clearly seen in SEM images which also supports FTIR analysis and thickness measurements after the tests.

**Figure 9:**
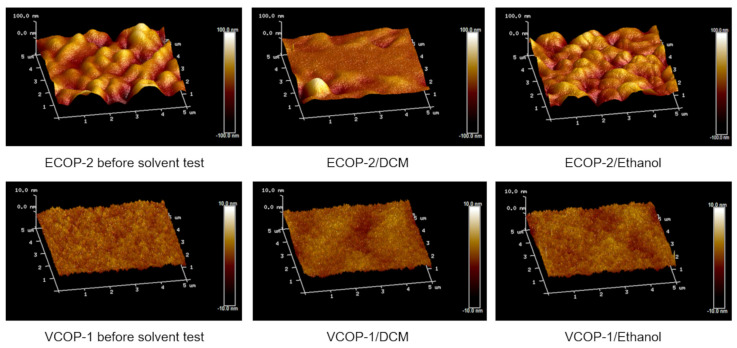
AFM surface analysis of copolymers before and after immersion in DCM and ethanol for 30 min.

Figure 9 shows AFM images of ECOP-2 and VCOP-1 copolymers before and after immersion in DCM and ethanol which support SEM analysis. For clarity, image scales are different as the VCOP-1 copolymer coating has a smoother surface. While the coatings had different surface morphologies, the change in surface roughness was clear for ECOP-2 copolymer while VCOP-1 copolymer showed no change in morphology in ethanol and exhibited the first signs of delamination in DCM.

The mean roughness (R_a_) and root-mean-square roughness (R_q_) values are also given in Table 2 below. As seen in Figure 9, the surface roughness of the ECOP-2 copolymer, both before and after the solvent test, is considerably higher than VCOP-1. In the literature, the existence of spherical structures leading to increased surface roughness on iCVD poly(GMA) films were reported [29,50]. However, both VCOP-1 and ECOP-2 copolymers contain GMA, and the VCOP-1 copolymer exhibits a very smooth surface. The difference between surface morphologies may be related solely to crosslinking monomer (EGDMA vs. V4D4). After immersion in solvents, surface roughness gradually decreases due to removal of ECOP-2 from the surface [65]. The VCOP-1 copolymer exhibited no significant change in surface roughness before the complete removal from the surface after 20 days.

### 3.4. Water and Saltwater Resistance

**Figure 10:**
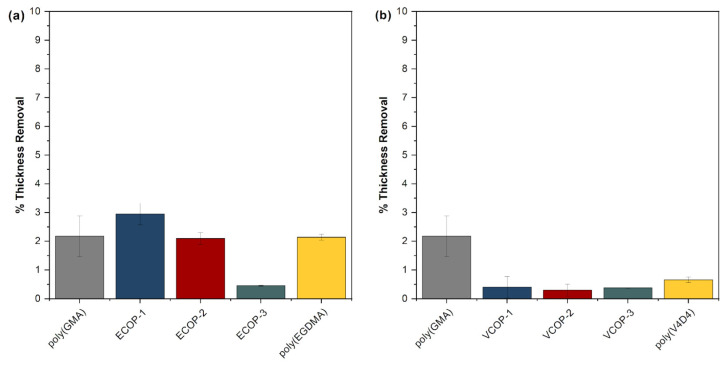
Relative change in film thickness for homo and (**a**) poly(GMA-co-EGDMA) (**b**) poly(GMA-co-V4D4) copolymer coatings immersed in water for 48 h.

Protective coatings play a vital role in electronic and optical devices providing a physical barrier between substrates and aggressive media. Electronic and optical devices can be exposed to water either by accident or intentionally due to the operational environment. iCVD polymers with some degree of crosslinking usually exhibit low solubility in water [40,51]. Water solubility tests were performed by immersion of iCVD homo- and copolymers in deionized water for 48 h. Thickness measurements performed before and after the water solubility test are given in Figure 10. Both poly(GMA) and poly(EGDMA) homopolymers showed around 2% thickness loss. Interestingly, only ECOP-3 copolymer showed significantly more resistance to immersion in water with less than 0.5% thickness loss. While poly(V4D4) homopolymer is resistant to immersion in water (around 0.5% thickness loss), all poly(GMA-co-V4D4) copolymers exhibited better resistance than homopolymers and ECOP copolymers.

**Figure 11:**
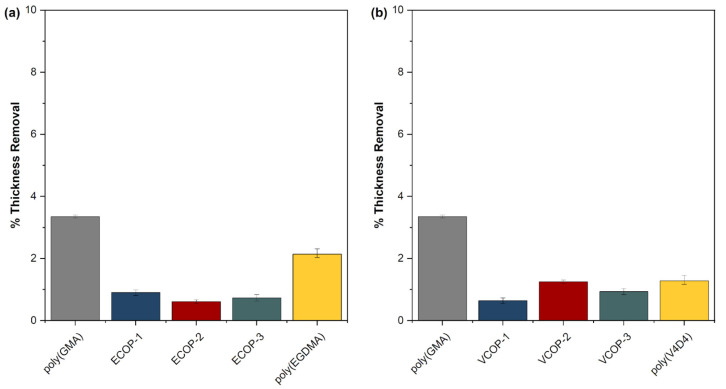
Relative change in film thickness of homo and (**a**) poly(GMA-co-EGDMA) (**b**) poly(GMA-co-V4D4) copolymer coatings immersed in 5 wt. % NaCl solution for 24 h.

Since optical surfaces can also be exposed to sea water, saltwater resistance tests of iCVD coatings on c-Si substrates were also performed. Coatings were immersed in 5 wt. % NaCl solution for 24 h. No significant changes in film thicknesses were observed, as shown in Figure 11. All coatings were quite resistant to the salt resistance test. All copolymers showed better saltwater resistance compared to homopolymer coatings with less than 2% thickness loss, demonstrating the effectiveness of crosslinking.

### 3.5. Adhesion Test

**Figure 12:**
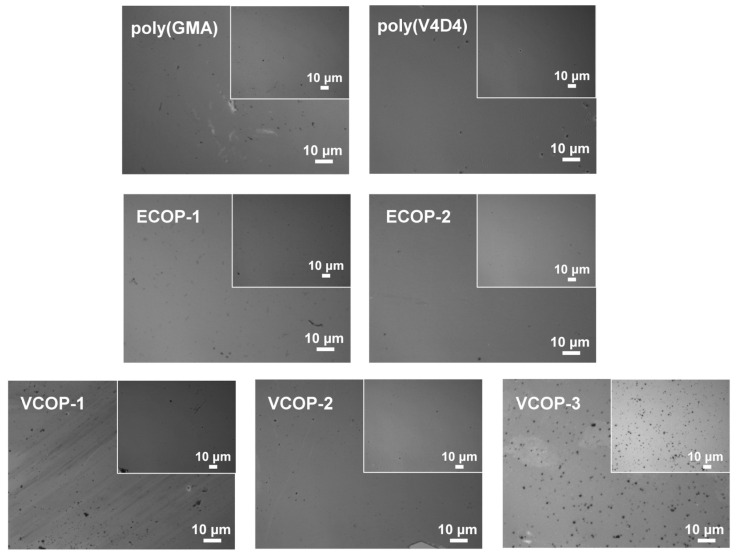
Optical microscopy images of homo and cross-linked copolymer films before (inset) and after (onset) adhesion test.

**Figure 13:**
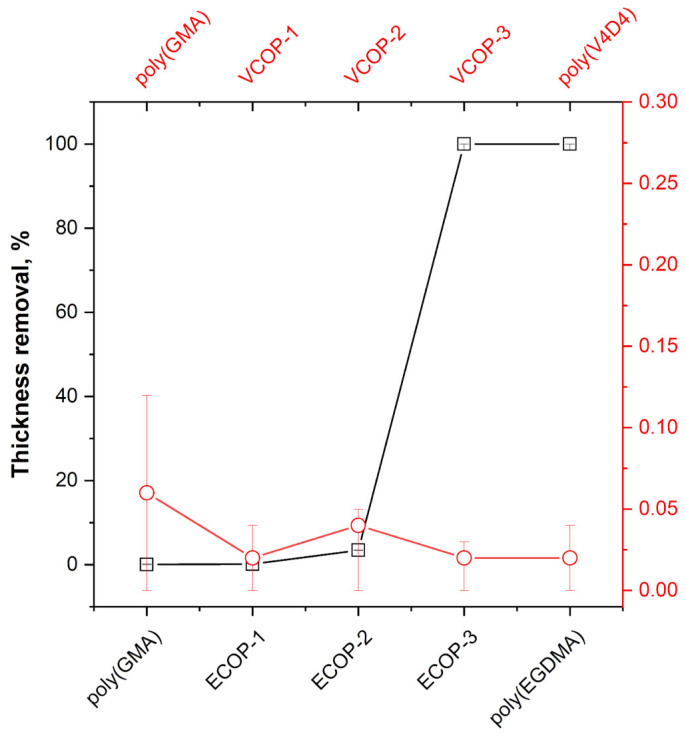
Relative change in film thickness of homo and copolymer coatings after the adhesion test (black squares-poly(GMA), poly(EGDMA) and ECOP copolymers; red circles-poly(GMA), poly(V4D4) and VCOP copolymers).

An ideal protective coating should have excellent chemical durability and good adhesion to the surface. To evaluate the adhesion of homo and copolymer iCVD coatings, standard adhesion tests were performed. Figure 12 shows the optical microscope images of coatings before and after an adhesion test. The large images (onset) in Figure 12 show the coating surface after the test with images in the upper right corner (inset) showing the pristine coatings. Cross-linked polymer coatings are known to have very good adhesion to most surfaces [51]. No defects or delamination were observed under optical microscope analysis of the surfaces for poly(GMA), ECOP-1 and ECOP-2. However, all sections of poly(EGDMA) and ECOP-3 coatings were removed by the cellophane tape from the surface during adhesion tests; hence, the optical microscope images of poly(EGDMA) and ECOP-3 are not given in Figure 12. MIL-F-1 48616 and MIL-C-48497A standards cover adhesion tests for optical coatings and consider the removal of less than 5% of the sample area acceptable. For poly(GMA), ECOP-1 and ECOP-2 coatings, the area removed during the test was less than 0.5% which is still within the acceptable range according to the test protocols followed, except for ECOP-3 and poly(EGDMA). Poly(V4D4) homopolymer and poly(GMA-co-V4D4) copolymer coatings successfully passed the adhesion tests. The only exception was VCOP-2 coating with a small area detaching from the surface. However, the area removed was less than 0.1% of the sample which is still less than the adhesion test limit of 5%. Relative change in film thickness of homo and copolymer coatings after the adhesion test are shown in Figure 13. Optical microscopy evaluation of the samples supports chemical durability test results indicating that poly(GMA-co-V4D4) copolymer coatings exhibit slightly better durability and adhesion than homopolymers and poly(GMA-co-EGDMA) copolymers.

**Figure 14:**
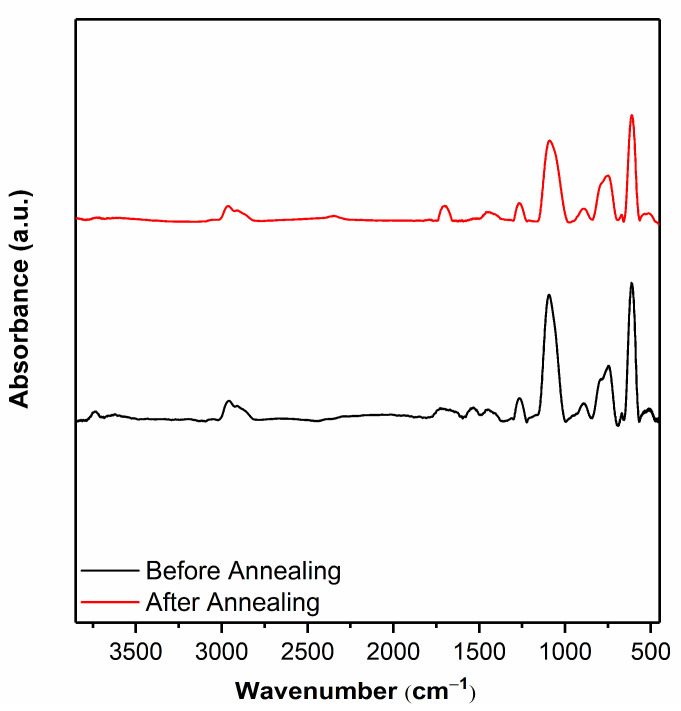
FTIR spectra of VCOP-1 films on c-Si substrate before and after annealing at 250 °C.

Optical devices are also exposed to temperature variations during operation. Coatings for optical surfaces should be stable in a wide temperature range. It has been shown that poly(GMA) thin films can be cross-linked via epoxy ring opening reaction by thermal annealing above 120 °C in air [47]. Thermal degradation and decomposition of poly(GMA) homopolymer film starts above 200 °C [50]. Cross-linking poly(GMA) polymers with a suitable monomer should increase chemical stability at higher temperatures. To evaluate temperature stability, VCOP-1 copolymer coatings were annealed at 250 °C. Although VCOP-1 copolymer exhibited up to 28% thickness loss during annealing, FTIR analysis revealed almost identical spectra before and after the test, indicating good chemical stability, as seen in Figure 14. The peaks assigned to asymmetric Si-O-Si stretching at 1090 cm^−1^ and C-O-C epoxy ring vibrations at 760 cm^−1^ remained clearly visible after the annealing test. However, the peak intensities of VCOP-1 coating slightly decreased after the test which might be related to the change in film thickness. The change in the film thickness may be partially related to relaxation of the copolymer film. Optical microscopy analysis of the coating surface after the analysis did not reveal significant changes, defects, or damage to the coating after annealing, confirming that crosslinked poly(GMA-co-V4D4) copolymer coatings are stable at temperatures up to 250 °C without any degradation.

**Figure 15:**
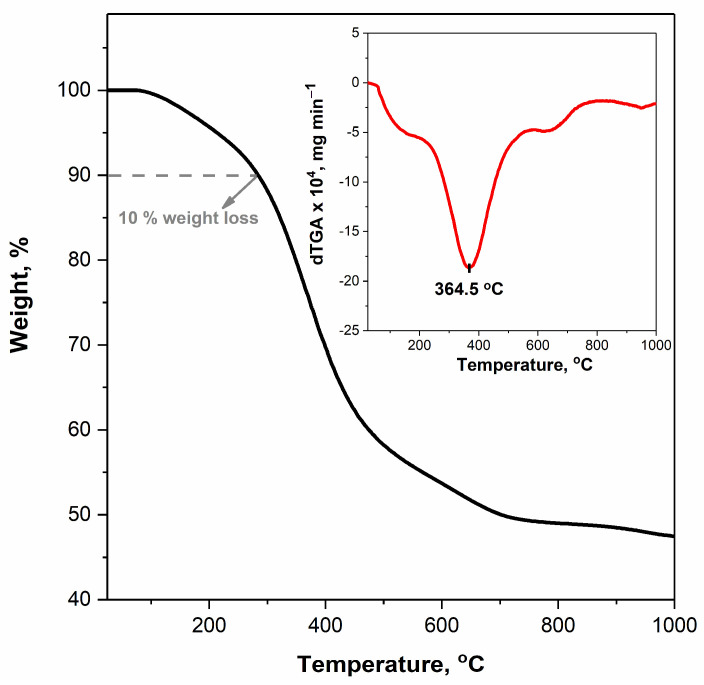
Experimental weight loss vs. T curves for thermal decomposition of VCOP-1 copolymer, black curve (insert, dTGA vs. T, red curve).

The thermal stability and degradation profiles of VCOP-1 were also evaluated by thermogravimetric analysis under nitrogen atmosphere, as shown in Figure 15. Siloxanes have high thermal stability due to the strength of their Si-O bond [66,67]. Therefore, the thermal stability of the copolymer produced using cross-linker (V4D4) is expected to be better than that of homopolymer poly(GMA) [68,69,70].

TGA curves shown in Figure 15 indicate that cross-linked VCOP-1 exhibits a degradation profile with three temperature zones which occur between room temperature to 177.8 °C, 285.7–473.4 °C and 580.6–709.3 °C, respectively. The first zone may be attributed to the loss of unreacted molecules during polymerization while the second and third weight losses correspond to the decomposition of the copolymer. Cross-linked VCOP-1 copolymer starts to decompose close to 285 °C and reaches the maximum decomposition temperature at 364.5 °C, and it has a second lower decomposition temperature at about 650.7 °C. The results are in good agreement with data for other GMA-based cross-linked copolymers in the literature [68,69]. TGA data show that the 10% weight loss temperature (T10%) is 285.7 °C which is considered to represent the beginning of degradation of the copolymer. When compared with the reported TGA data of homopolymers poly(GMA) [70] and poly(V4D4) [67] in the literature, crosslinked VCOP-1 has better thermal stability than uncrosslinked poly(GMA) homopolymer.

### 3.6. Optical Transmittance

Unlike most protective coatings used in a variety of substrates such as metals, plastics, electronic components, etc., protective coatings for optical surfaces must be transparent in the wavelength of interest and they must not affect the optical performance of the coated surface.

**Figure 16:**
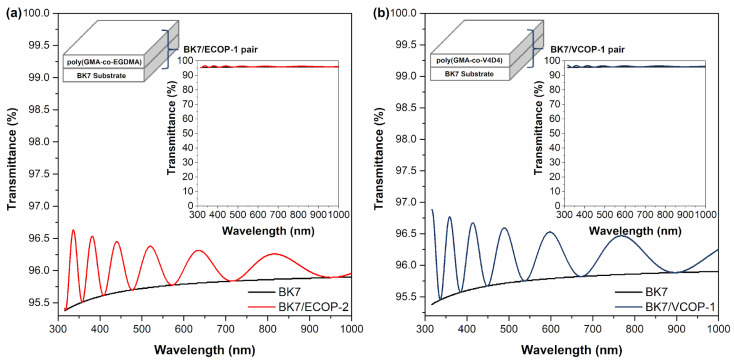
Modeled optical transmittance of (**a**) BK7/ECOP-2 and (**b**) BK7/VCOP-1 copolymer coatings.

Optical transmittance of selected iCVD copolymers on BK7 glass substrates was modeled using TF Companion software between 300 and 1000 nm range. BK7 is a high-quality optical glass with high optical transmittance in the visible and near IR spectrum and is the most commonly used glass for optical windows, lenses, and prisms. In previous studies, it was shown that a coating with a thickness of less than 1000 nm would not significantly affect the optical transmittance of BK7 glass substrate in the visible region [50]. The simulated optical transmittance of 1 μm thick ECOP-2 and VCOP-1 copolymer on BK7 glass substrates are shown in Figure 16. Simulated optical transmittance of ECOP-2 was slightly lower than VCOP-1 due to its higher refractive index (*n* = 1.495) of VCOP-1 (*n* = 1.487); however, the difference is negligible. Simulation results show that both copolymer coatings should not change the optical transmittance of BK7 glass substrate.

**Figure 17:**
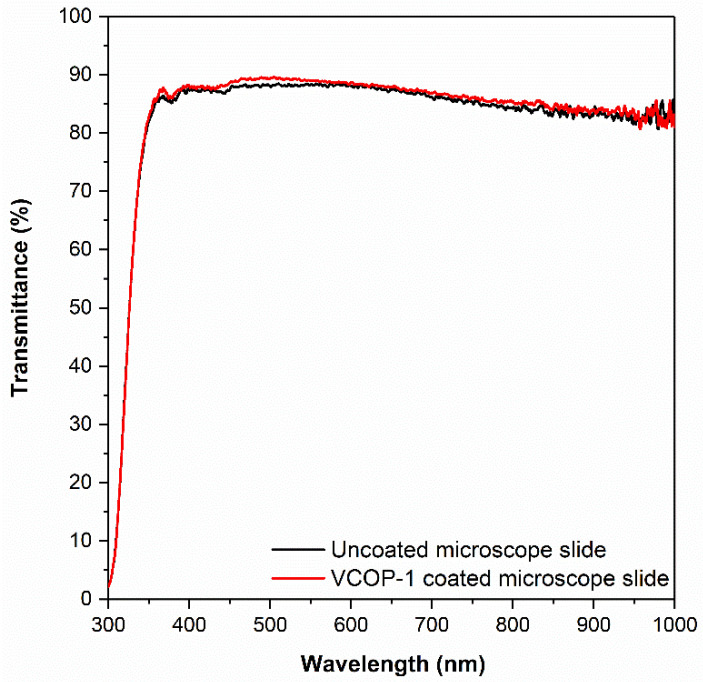
Measured optical transmittance of uncoated and VCOP-1 coated glass substrates.

To confirm the simulation results, 400 nm thick VCOP-1 copolymers were deposited on optical glass substrates via iCVD using the conditions given in Table 1. The optical transmittance of VCOP-1 coated, and uncoated BK7 glass substrates are shown in Figure 17. Experimental measurements confirm simulation results. Although uncoated BK7 glass showed much lower optical transmittance (>85% in visible spectrum) compared to simulated transmittance (~95–96%), 400 nm VCOP-1 copolymer coating did not reduce the optical transmittance, it increased the transmittance very slightly between 450 and 600 nm. The difference between simulated and measured optical transmittance for uncoated and coated glass substrates is mainly due to the quality of glass substrates used in the experimental study.

It should be noted that in the iCVD process the deposition rate of the polymer film strongly depends on the substrate temperature and does not depend on the substrate material; however, the final film morphology is affected by the substrate surface and film thickness. No measurable differences between chemical stability, optical transmittance and adhesion were observed between 350–400 nm thick iCVD copolymers deposited on glass and c-Si. 

## 4. Conclusions

In this work, thin (350–400 nm) poly(GMA), poly(EGDMA), poly(V4D4) homopolymers and copolymers of poly(GMA-co-EGDMA) (ECOP series) and poly(GMA-co-V4D4) (VCOP series) with varying compositions were synthesized via the iCVD process on c-Si and glass substrates. The chemical durability, thermal stability, optical transmittance, and adhesion of coatings were evaluated for protection of optical surfaces used in a variety of applications. A comparative study has been carried out between ECOP and VCOP series of copolymer coatings. FTIR analysis confirmed that cross-linked copolymer thin films with the desired chemical composition can be obtained simply by changing the monomer flow rates during the polymerization process. It was also confirmed that functional groups of monomers are preserved in the process. ECOP-2 and VCOP-1 coatings were found to be the better performing coatings after chemical durability tests performed using various solvents. The durability of OP-1 coating was better than ECOP-2, especially in organic solvent immersion, water solubility and long-term stability tests, due to V4D4 cross-linker. VCOP-1 copolymer showed high thermal resistance and it was chemically stable at 250 °C. 400 nm thick VCOP-1 copolymer coating on glass exhibited excellent optical transparency in the visible spectrum and did not affect the optical performance of the glass substrate. The results also show that non-fluorinated polymers can be tailored via iCVD copolymerization to fabricate protective transparent coatings for optical surfaces. The findings indicate that poly(GMA-co-EGDMA) and poly(GMA-co-V4D4) copolymer thin film coatings can provide excellent chemical and physical protection from the elements for optical surfaces without sacrificing optical performance. In theory, the chemical durability of the coatings could be further improved with addition of a fluorinated monomer, if necessary. The iCVD process also enables the preservation of functional groups during polymerization, so additional functionalities can be further activated. The possibility of removing the copolymer coatings using a suitable solvent and re-coating the surface is a big advantage of iCVD polymer coatings over inorganic coatings that are currently being used. In addition, the advantage of scalability, the ability to control film morphology and the ability to conformally coat complex non flat optical surfaces make the iCVD process an excellent candidate for fabrication of protective coatings at low cost. 

## Figures and Tables

**Table 1:** Summary of iCVD process conditions for copolymers.

iCVD Sample	Substrate Temp. °C	Filament Temp. °C	Pressure mTorr	Flow Rate, sccm				Flow Ratio Crosslinker/ GMA
				TBPO	GMA	EGDMA	V4D4	
poly(GMA)	25 or 35	300	250	0.8	0.4			
ECOP-1	25	330	515	2	1.6	0.4		0.25
ECOP-2				2	0.8	0.4		0.5
ECOP-3				2	0.4	0.4		1
poly(EGDMA)	25	330	515	2		0.4		
VCOP-1	35	300	250	0.8	0.4		0.1	0.25
VCOP-2				0.8	0.4		0.2	0.5
VCOP-3				0.8	0.4		0.4	1
poly(V4D4)	25	300	250	0.8			0.4	

**Table 2:** Surface roughness changes for ECOP-2 and VCOP-1 copolymer coatings.

Sample	R_q_	R_a_
ECOP-2	23.1	17.3
ECOP-2/Ethanol	17	12.5
ECOP-2/DCM	9.46	7.25
VCOP-1	0.807	0.646
VCOP-1/Ethanol	0.756	0.6
VCOP-1/DCM	0.931	0.743

## Data Availability

Not applicable.
